# Electrical Stimulation of Lateral Habenula during Learning: Frequency-Dependent Effects on Acquisition but Not Retrieval of a Two-Way Active Avoidance Response

**DOI:** 10.1371/journal.pone.0065684

**Published:** 2013-06-28

**Authors:** Anton Ilango, Jason Shumake, Wolfram Wetzel, Henning Scheich, Frank W. Ohl

**Affiliations:** 1 Department Systems physiology of learning, Leibniz Institute for Neurobiology, Magdeburg, Germany; 2 Institute of Biology, Otto-von-Guericke University, Magdeburg, Germany; 3 Department of Psychology, The University of Texas, Austin, Texas, United States of America; 4 Center for Behavioral Brain Science, Otto-von-Guericke University, Magdeburg, Germany; Medical University of South Carolina, United States of America

## Abstract

The lateral habenula (LHb) is an epithalamic structure involved in signaling reward omission and aversive stimuli, and it inhibits dopaminergic neurons during motivated behavior. Less is known about LHb involvement in the acquisition and retrieval of avoidance learning. Our previous studies indicated that brief electrical stimulation of the LHb, time-locked to the avoidance of aversive footshock (presumably during the positive affective “relief” state that occurs when an aversive outcome is averted), inhibited the acquisition of avoidance learning. In the present study, we used the same paradigm to investigate different frequencies of LHb stimulation. The effect of 20 Hz vs. 50 Hz vs. 100 Hz stimulation was investigated during two phases, either during acquisition or retrieval in Mongolian gerbils. The results indicated that 50 Hz, but not 20 Hz, was sufficient to produce a long-term impairment in avoidance learning, and was somewhat more effective than 100 Hz in this regard. None of the stimulation parameters led to any effects on retrieval of avoidance learning, nor did they affect general motor activity. This suggests that, at frequencies in excess of the observed tonic firing rates of LHb neurons (>1–20 Hz), LHb stimulation may serve to interrupt the consolidation of new avoidance memories. However, these stimulation parameters are not capable of modifying avoidance memories that have already undergone extensive consolidation.

## Introduction

Several neural systems implicated in reward-oriented approach are also involved in the avoidance of negative outcomes (e.g. [1], [2]), and release of dopamine (DA) plays a role in both types of motivational processes [3], [4]. Among these systems, the lateral habenula (LHb) is functionally coupled to the dopaminergic systems during motivated behavior to signal negative affective states such as reward omission and punishment prediction [5].

The LHb is an epithalamic structure containing glutamatergic neurons involved in various processes such as the regulation of sleep, negative reward prediction, stress, learned helplessness, depression and behavioral inhibition [6], [7], [8], [5]. The LHb receives input from regions involved in motivated behaviors, such as the internal segment of the globus pallidus (entopeduncular nucleus in rodents), ventral pallidum, ventral striatum, and lateral hypothalamus. The LHb modulates dopaminergic and serotonergic neurons, which project to LHb input regions and also reciprocate direct projections with the LHb [9], [10], [8]. Thus, the LHb is anatomically positioned as a relay station between forebrain limbic-motor circuits and brainstem monoaminergic systems, and would appear to be a key node for rapidly influencing the selection of behavioral responses during learning [11], [12], [9], [13], [8].

LHb neurons are activated by aversive stimuli and express elevated c-fos following pain [14], [15]. Of particular relevance are the seminal findings by Hikosaka and colleagues that LHb neurons are also activated by the absence of expected reward, thus generating a negative error signal in reward prediction [16]. Human imaging studies have also shown increased blood flow to the habenula region in response to negative feedback following failed trials in a perceptual-motor learning task [17], [18] and Granger causality analysis indicated a causal influence from the habenula to the ventral tegmental area (VTA) during these trials [18]. Moreover, several studies have reported that electrical stimulation applied to the LHb inhibits the majority of dopaminergic neurons in rodents [19], [20] and primates [16] mainly through the rostromedial tegmental nucleus [21], [22]. Since the activation of dopaminergic neurons is known to facilitate approach by sending a behavioral “go” signal [23], a postulated role for the LHb may be to provide an opponent “stop” signal that suppresses motor activity [8].

Even though LHb neurons are known for promoting withdrawal behavior in response to the absence of reward or presence of aversive stimuli [16], [24], their role in avoidance learning is less explored. In active avoidance learning, an animal first learns to predict a conditioned stimulus (CS) signaling arrival of an aversive unconditioned stimulus (US) while also learning to escape the US by performing an operant response. The animal then learns to initiate this escape response during the CS interval, thus avoiding exposure to the US entirely. Previous studies indicated that chemical lesions applied to the LHb showed either no effect on the acquisition of one-way active avoidance [25], or a detrimental effect if the task was made exceptionally difficult and stressful [26]. On the other hand, under paradigms of two-way active avoidance, lesioning the habenula led to either a robust facilitative effect [27] or limited facilitative effect (specifically associated to the second acquisition session) depending on task procedures [28]. Facilitation by LHb lesion during a more cognitively demanding task such as two-way active avoidance might be due to increased DA release in the nucleus accumbens [29] and prefrontal cortex [30], which are known to strengthen the stimulus-response associations underlying the acquisition of avoidance learning [3], [31]. Moreover, rats selectively bred for poor escape learning show extremely elevated metabolic activity in the LHb and impoverished metabolic activity in the VTA [32], [33], which may be explained by increased excitatory LHb inputs, specifically on neurons projecting to the VTA [34].

All of this evidence suggests that activation of the LHb may bias an animal toward passive, “no go” behaviors at the expense of active, “go” behaviors, particularly when the correct “go” response is ambiguous or difficult to acquire. This is the case for two-way active avoidance learning, in which there is a conflict between avoiding shock and entering a place previously paired with shock. Evidence has shown that acquisition of two-way active avoidance learning is made easier when feedback stimulation is applied to the VTA or lateral hypothalamus during the affective “relief” state following successful avoidance [35], [36], [37]. We further hypothesized that LHb stimulation would have the opposite effect and exacerbate the go vs. no-go conflict in the early stages of two-way active avoidance learning, leading to retarded acquisition. Not only did LHb stimulation retard acquisition, it apparently led to the consolidation of an ambivalent response strategy (a mixture of avoidance and escape), which did not improve when stimulation was discontinued, even after five days of additional training [35].

This previous study used a stimulation frequency of 100 Hz in order to match the stimulation frequency optimized for rewarding brain simulation of the VTA, which we wished to compare directly with the effects of LHb stimulation. However, it is unknown whether the effect on learning was frequency-dependent or whether it might be evident at lower frequencies. Studies showing suppression of VTA firing from LHb stimulation in the rat used very low frequencies (10 Hz for Christoph et al. [19] and 0.5 Hz for Ji and Shepard [20]). However, these were anesthetized preparations. In awake animals, studies have typically used higher frequencies of brain region stimulation as feedback for reinforcement occurrences (e.g., [38], [39]). Specifically in the case of the LHb, Matsumoto et al. [39] used 300 Hz stimulation as post-saccadic feedback to achieve behavioral effects in monkeys. However, 130 Hz stimulation was reported to disrupt, not enhance, LHb function in rats [34]. This is most likely because Li et al [34] delivered LHb stimulation continuously for an hour (to mimic deep-brain stimulation protocols used in human patients) as opposed to the brief, intermittent stimulation used by us and by Matsumoto et al. [39] in conjunction with learning trials. Nonetheless, behavioral effects at lower frequencies would strengthen the argument that the disruption in avoidance learning is due to an activation of the LHb and not several ambiguous effects associated with the high frequency stimulation [40], [41], [42], [43], [44].

Using slice recordings Chang and Kim [45] observed spontaneous, tonic firing rates in LHb neurons between 1–20 Hz, with a brief hyperpolarizing current leading to bursts in the 80–90 Hz range. In anesthetized preparations, three different spontaneous firing patterns were reported: regular tonic, irregular tonic, and regular bursts with a mean firing rate of 12.8 Hz [46]. To our knowledge, only one study has recorded firing rates of LHb neurons in awake, behaving rats: Sharp et al. [47] reported a typical LHb neuron firing at 17 Hz when the rat was at rest and at 40 Hz when the rat was in motion. Based on these findings, we explored the effects of three frequencies: a low frequency (20 Hz) similar to tonic firing at rest, a high frequency (100 Hz) similar to a firing burst, and an intermediate frequency (50 Hz) similar to that observed during active exploration [47].

Overall, seven different groups of animals were used. Three of these groups received stimulation (20, 50, or 100 Hz) during the acquisition phase (first five sessions) and no stimulation during the retrieval phase (last five sessions), and the other three groups received stimulation (20, 50, or 100 Hz) during the retrieval phase, but no stimulation during the acquisition phase. For these groups, only frequency was varied while train duration, intensity, and pulse duration were held constant [48], [49]. This allowed us to investigate the effect of temporal integration of different frequencies of stimulation within the avoidance or “relief” phase. However, this also led to a potential confound, in that each group received a different number of total pulses during each stimulation train: 4, 10, and 20 pulses for the 20, 50, and 100 Hz groups, respectively. Therefore, based on initial data showing an effect of 50 Hz but no effect of 20 Hz, we added an additional 20 Hz group that received the same number of pulses as the 50 Hz group, but spread out over 500 ms instead of 200 ms, in order to determine whether frequency or number of pulses was the more relevant factor. Similar to our previous studies, we used Mongolian gerbils, which are a suitable animal model to study motivated behavior, particularly with an auditory learning component [50], [51], [52], [53], [35], [36], [37].

## Materials and Methods

### Subjects

A total of 72 adult male Mongolian gerbils (*Meriones unguiculatus*) obtained from Tumblebrook Farms, West Brookfield, MA, USA (age: 3–6 months, weight: 76 g–98 g) were used in this study. Gerbils were individually housed 3 days before experiments started and maintained on a 12 h light/dark cycle (light on 07:00–19:00h) throughout the experiment. Experiments were conducted between 07:00 to 17:00 h, with individual subjects being trained at a consistent time of day to maintain their daily session interval. All experimental procedures were approved by the Ethics Committee of the State of Sachsen-Anhalt, Germany.

### Surgical Procedures

Surgery and implantation of electrodes were performed under ketamine (100 mg/kg) and xylazine (5 mg/kg) anesthesia. Animals were fixed in a stereotaxic frame (David Kopf Instruments, USA). Bipolar stimulation electrodes with the tips separated by ∼0.2 mm were custom made from Teflon-insulated stainless steel microwires (diameter: with Teflon: 140 µm; without Teflon 75 µm; Science Products GmbH, Germany) and implanted at the level of the LHb (1.6 mm posterior to bregma, 0.6 mm lateral to the midline, 2.6 mm ventral to the brain surface) with the incisor bar set at −4.0±0.5 according to the stereotaxic atlas for gerbil by Loskota [54]. Due to the relatively large anterior-posterior extent of the LHb, the poles of the bipolar electrodes were aligned rostro-caudally. The electrode was fixed in place with dental acrylic cement, and half of the animals were implanted in the right hemisphere and the other half in the left.

### Shuttle Box Avoidance Learning: Basic Procedures

Two-way active avoidance learning was investigated using shuttle-box conditioning procedures similar to that described previously [35], [37]. The shuttle-box (38 cm×19 cm×22.5 cm) had two compartments separated by a 6 cm high hurdle. Each daily session consisted of 60 trials with a variable intertrial interval (ITI) of 20–24 s. A session began with a 3 min habituation period. The CS was a series of 2 kHz pure tones (6 s, 200 ms tone duration, 300 ms inter-tone interval, 65–70 dB SPL). The US was a foot shock applied through the grid floor at the end of the CS in case the animal did not cross the hurdle during the 6 s CS presentation. The US was switched off when the animal escaped, i.e., crossed the hurdle during US presentation, or after a maximum duration of 4 s if the animal failed to escape. The intensity of the US was slowly raised from 400 µA to 600 µA during the first training session. Since the rate of conditioning also depends on the change in US intensity, we subsequently maintained a constant US intensity of 600 µA. Crossing the hurdle during the tone presentation was considered the conditioned response (CR). Successful performance of the CR terminated the CS and preempted the US, i.e., resulted in avoidance of shock. A flexible cable connected with a swivel allowed for electrical brain stimulation and easy movement during shuttle-box learning.

### Details of Stimulation Procedures and the Experimental Groups

Three different frequencies of LHb stimulation were investigated. Stimulation was delivered either during acquisition (session 1–5) or during the retrieval phase (session 6–10) immediately following each successful avoidance response. In each case, three different groups with different frequencies were employed: 20 Hz, 50 Hz, and 100 Hz. Across all stimulation frequencies, the train duration (200 ms), intensity (100 µA), and duration of pulses (biphasic pulses of 0.2 ms) were held constant. This meant that the number of pulses varied with frequency: 1) the 20 Hz group received 4 pulses of 0.2 ms separated by an inter pulse interval (IPI) of 50 ms; 2) the 50 Hz group received 10 pulses separated by an IPI of 20 ms; and 3) the 100 Hz group received 20 pulses separated by an IPI of 10 ms. We did not alter the current intensity or pulse duration since increasing both would have increased the spatial size of the field of stimulation, and the constant train duration was necessary to limit stimulation to the time window of reinforcement occurrence. To address the confound that pulse number rather than frequency might have been responsible for differences observed between the 20 and 50 Hz groups, we ran an additional group that received the same number of pulses as the 50 Hz group (10 pulses) spread out over a larger window of time (500 ms). Data for the 100 Hz group were previously published [35] but reanalyzed here for determination of a frequency-response curve.

### Data Analysis

The success rate (number of successful avoidance responses divided by total number of trials), average latency to initiate response (with failed trials assigned a latency score of 6 s, the maximum length of the CS), and ITI crosses (spontaneous hurdle crosses during the ITI) were calculated for each session. Success rate and response latency were used as measures of avoidance learning, and ITI crosses were used as a measure of non-specific motor activity. These data were analyzed using repeated measures ANOVAs, with frequency of stimulation (no stimulation, 20 Hz, 50 Hz, 100 Hz) as a between-subjects variable and training session as a within-subjects variable. Directional Dunnett post hoc tests were used to test the hypothesis that each stimulated group would have a lower rate of successful responding and higher response latencies compared to implanted, non-stimulated controls. The effect of discontinuing stimulation was evaluated using a planned comparison of session 5 (the final training session with stimulation) with session 10 (the final training session without stimulation). A Pearson product-moment correlation (*r*) was also calculated to assess the potential relationship between avoidance performance and general motor activity, as measured by spontaneous hurdle crossing during the ITI. Original data of the learning experiments can be made available upon request to the authors.

### Histology

**Figure 1:**
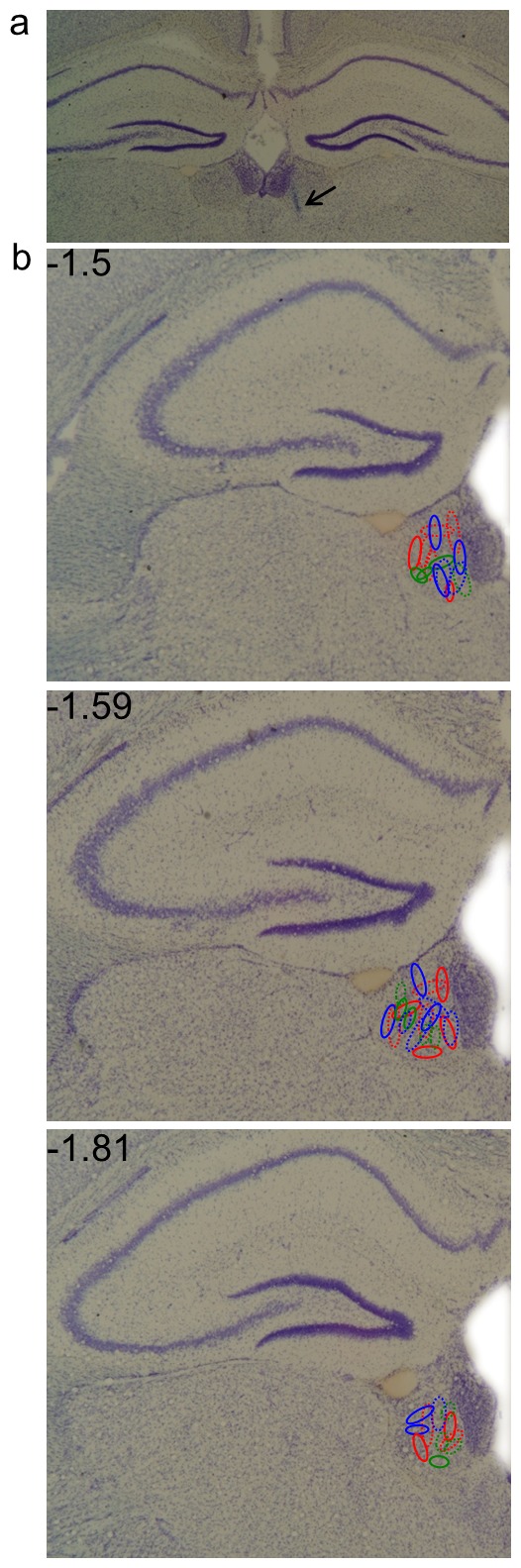
Localization of electrode positions in the LHb. a) Section showing representative example of Cresyl violet and Prussian blue staining. Arrow indicates electrode position. b) Oval shaped symbols illustrate all electrode positions. Dashed oval symbols indicate subjects which received stimulation after acquisition. Green, blue and red colours indicate 20, 50 and 100 Hz stimulation respectively.

At the conclusion of behavioral experiments, the gerbils were deeply anesthetized with a ketamine-xylazine mixture. Thereafter, the animals were decapitated. The brains were rapidly isolated and frozen in isopentane immersed in liquid nitrogen and finally stored at −20°C. Subsequently, 40 µm coronal sections from 0.5 mm to 3.5 mm posterior to bregma were obtained using a sliding microtome (Leica cryostat). Nissl staining combined with Prussian blue was performed to reveal the ion deposits around the electrode tips (Figure 1). The locations of the electrode tips were determined with reference to the stereotaxic atlas of gerbils [54]. Only subjects with correct electrode placements within the LHb were included in the data analysis (N = 53).

## Results

### Stimulation during acquisition (session 1–5)

**Figure 2:**
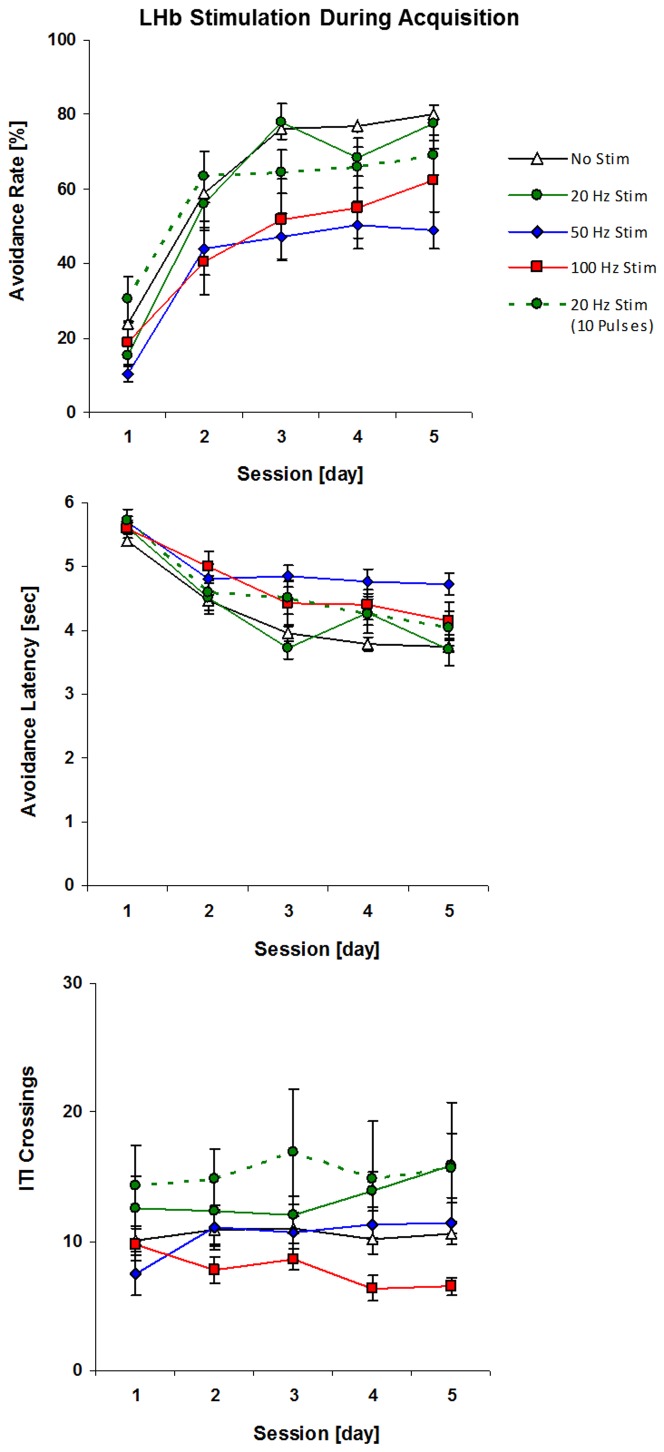
Effect of LHb stimulation on acquisition. Sample size was 22 for the no stimulation group (data from first five sessions were pooled from all the groups which received stimulation during last five sessions), 6 for 20 Hz group, 8 for 50 Hz group and 8 for 100 Hz group. These groups (indicated by solid lines) were stimulated for 200 ms immediately following each successful response. In addition, we included a separate control group (indicated by dashed line, n = 8) which was stimulated at 20 Hz for 500 ms, i.e., the same frequency as the 20 Hz group but the same number of pulses as the 50 Hz group. Only the 50 and 100 Hz groups showed learning curves that were significantly different from the no-stimulation group after post hoc correction (*p*<.05).

The avoidance data were analyzed with a 4×5 (stimulation×session) repeated measures ANOVA. The 4 levels of stimulation were no stimulation, 20 Hz, 50 Hz, and 100 Hz, and sessions 1–5 served as the repeated measure. For both avoidance responding and latency, there was a significant main effect of session, *F*(4,164)>77.2, *p*<.001, and a significant main effect of stimulation, *F*(3,41)>6.94, *p*<.001. However, these effects were of little interest in light of the significant two-way interaction (stimulation×session) obtained for rate of successful avoidance responses, *F*(12,164) = 2.13, *p* = .02. This interaction term reflects a significant difference in the slopes of the learning curves across groups, with animals receiving 50 Hz or 100 Hz achieving lower levels of avoidance responding than animals receiving 20 Hz or no stimulation (Figure 2, top panel). A similar pattern of results was found for the measure of avoidance latency, with a highly significant two-way interaction, *F*(12,164) = 3.0, *p* = .001, reflecting poorer acquisition curves in the 50 and 100 Hz groups relative to the 20 Hz and no stimulation groups (Figure 2, middle panel). Dunnett post hoc comparisons indicated that both 50 and 100 Hz groups were impaired relative to the no-stimulation group (*p*<.01 for both groups), but there was no significant difference for the 20 Hz group (*p*>.50).

### Stimulation during retrieval (session 6–10)

**Figure 3:**
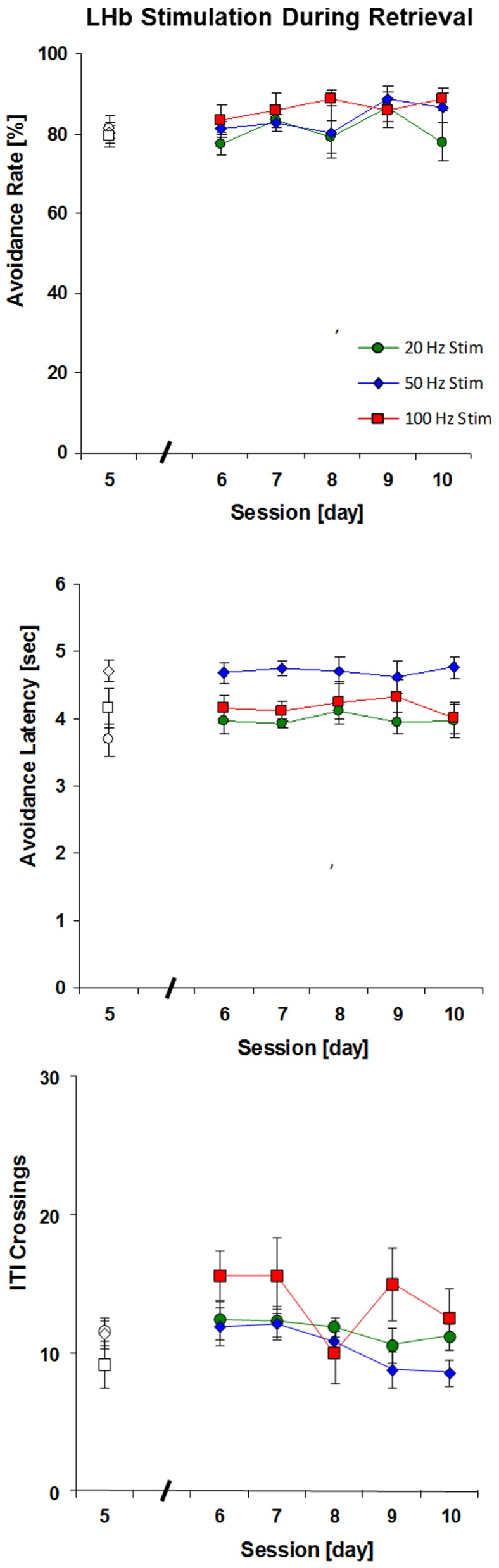
Effect of LHb stimulation on retrieval i.e., last five sessions. Open symbols indicate baseline performance before stimulation was initiated.

To further address the question of whether the effect of LHb stimulation reflected an impairment of learning versus a direct suppression of avoidance behavior, a group implanted with LHb electrodes was trained initially without stimulation until learning approached the asymptote of 80–90% successful responding after five sessions. Then LHb stimulation was given for five additional training sessions. These data were analyzed with a 3×6 (stimulation×session) repeated measures ANOVA. The six sessions included the fifth session of acquisition as a baseline without stimulation followed by sessions 6–10 with 20, 50, or 100 Hz stimulation. In terms of successful avoidance responses, no decrement in performance was observed during these sessions (Figure 3); in fact, there was continued improvement in performance for all groups with a significant main effect of session, *F*(5,95) = 2.59, *p* = .03. Frequency of stimulation had no impact on behavior, either as a main effect, *F*(2,19) = 0.84, *p* = .45, or as an interaction with session, *F*(10,95) = 1.19, *p* = .31. A similar pattern was observed for the response latency data, with a significant main effect of session, *F*(5,95) = 2.91, *p* = .02, but no significant main effect of stimulation, *F*(2,19) = 3.02, *p* = .07, or interaction, *F*(10,95) = 0.46, *p* = .91.

### Discontinuation of stimulation (session 6–10)

**Figure 4:**
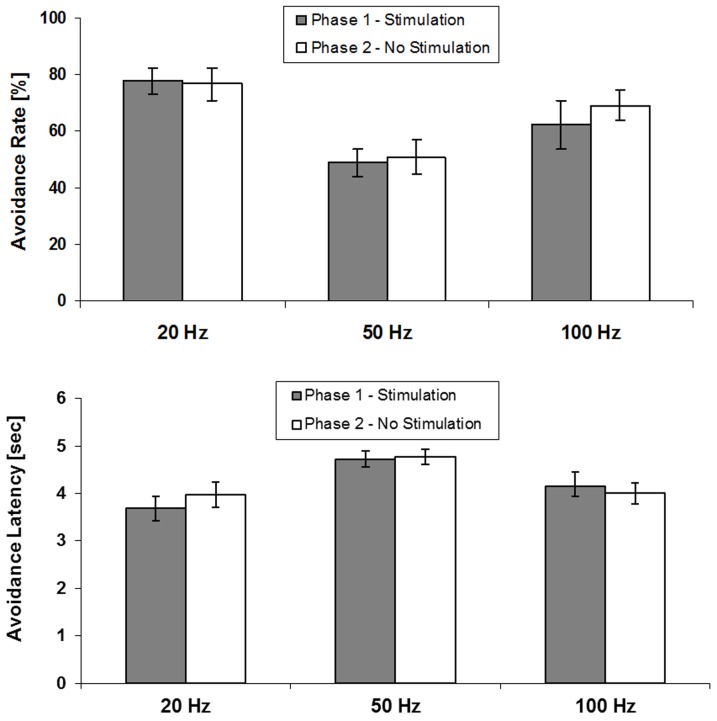
Mean ± SEM of percentage of avoidance rate and avoidance latency in session 5 with LHb stimulation and in session 10 after 5 additional training sessions without stimulation.

Paired-samples t tests were used to compare session 5 (the final training session with stimulation) vs. session 10 (the final training session without stimulation). As illustrated in Figure 4, there was no significant change in avoidance performance (measured by successful responses and response latencies, respectively) for the 20 Hz group (*p* = .90 and .59), 50 Hz group (*p* = .70 and .83), or the 100 Hz group (*p* = .50 and .65).

### Effect of stimulation on motor activity

To analyze potentially confounding effects of LHb stimulation on general motor activity, we quantified the number of hurdle crossings during the ITI, i.e., spontaneous crossings that were not evoked by the CS or the US. The total ITI crossings per session were evaluated with a 4×5 (stimulation×training session) repeated-measures ANOVA. The main effect of stimulation did not reach significance, *F*(3,41), *p* = 0.06, but there was a non-significant trend for slightly reduced activity in the 100 Hz group and slightly increased activity in the 20 Hz group. There was no main effect of session *F*(4,164) = 0.61, *p* = .66, and no significant interaction *F*(12,164) = 1.4, *p* = .17. Moreover, there was no relationship between ITI crosses and successful avoidance responses, *r*(45) = .02, *p* = .88, indicating that the observed effects on avoidance learning cannot be attributed to general differences in motor activity.

### Effect of frequency vs. pulse number on acquisition

To test whether 50 Hz frequency (receiving 10 pulses within 200 ms of making the operant response) was the critical parameter for impairing acquisition rather than total pulse number, we tested an additional 20 Hz group that received the same total number of pulses as the 50 Hz group (10 pulses within 500 ms of the operant response). This allowed us to test the effect of pulse number while holding frequency constant. These avoidance data were analyzed with a 3×5 (stimulation×session) repeated measures ANOVA. The 3 levels of stimulation were no stimulation, 4 pulses, or 10 pulses per 20 Hz stimulation train. A two-way interaction (stimulation×session) for rate of successful avoidance responses was significant, *F*(8,132) = 2.49, *p* = .02. The interaction reflected that performance gains were similar for all groups between the first two sessions, but tended to level off thereafter for the group that received the most pulses. However, this interaction term did not approach significance for the measure of avoidance latency, *F*(8,132) = 1.32, *p* = .24, and the main effect of stimulation was not significant for either responding, *F*(2,33) = 0.45, *p* = .64, or latency, *F*(2,33) = 1.11, *p* = .34. Dunnett post hoc comparisons indicated that neither success rate nor response latency of the 10 pulse/20 Hz group was significantly different from no-stimulation control (*p* = .35 and .13, respectively).

## Discussion

The results support a disruptive effect of LHb activation on negative reinforcement, with consequences for acquisition, but not retrieval, of an active avoidance response. Overall, the results of 50-Hz stimulation replicate our previous findings using 100-Hz stimulation [35] and appear to have an even stronger disruptive effect on acquisition processes while still having no discernible effect on retrieval processes. However, this effect was not observable at 20 Hz—regardless of whether duration or pulse number was held constant with the 50 Hz group—perhaps because this frequency falls within the spontaneous, tonic firing rate of LHb neurons [45], [47]. Notably, there were no effects of LHb stimulation on motor activity during the ITI. Since animals were only stimulated immediately *after* an avoidance response and there was no evidence of lingering motor effects before the next trial, a motor mechanism cannot explain the observed decrement in avoidance acquisition. In addition, there was a significant interaction effect between stimulation and time, indicating the *rate* of acquisition was slowed. This suggests a learning mechanism.

Analogous results were obtained in a positive reinforcement paradigm by Matsumoto and Hikosaka [39], who stimulated the LHb of monkeys following an eye saccade to a rewarded target using similar parameters to ours: namely, brief trains of stimulation during the reinforcement interval. LHb stimulation was found to suppress saccades in the same manner that reward omission does. This finding and our present results suggest that the LHb is capable of discounting signals of reinforcement, both positive and negative. However, unlike our results, Matsumoto and Hikosaka [39] showed that LHb stimulation suppressed saccades *after* they had been acquired, suggesting a potential role of the LHb in behavioral extinction or reversal of learned behavior. In contrast, we did not observe such an effect on avoidance behavior when LHb stimulation was started late in training. That is, LHb stimulation could suppress the acquisition of an avoidance response, but it could not suppress an avoidance response once it was learned. The discrepancy may be due to differences in stimulation and training parameters. Perhaps with a stronger activation of the LHb (e.g., the 300 Hz frequency used in their study) or with a weaker consolidation of the response (e.g., initiating stimulation before learning becomes asymptotic), LHb stimulation could elicit “unlearning” of an avoidance response as well. However, this may also be a point where the neural mechanisms of positive and negative reinforcement part company. Unlike rewarded behavior, avoidance behavior is not maintained by continued exposure to the unconditioned stimulus, which, in the late stages of avoidance learning, is minimal. This self-sustaining nature of avoidance behavior renders it unusually persistent once it is acquired [55]. Thus, in the late stages of learning, positively reinforced behavior may be more malleable and negatively reinforced behavior may be more resistant to the effects of LHb stimulation.

What is perhaps surprising is the long-term avoidance deficit induced by LHb stimulation. As was the case with 100-Hz stimulation [35], the behavioral impairment resulting from five training sessions with 50-Hz stimulation was still evident after five additional training sessions without stimulation. The mechanism for this long-term effect is unclear, but it bears a striking resemblance to the phenomenon of learned helplessness, in which exposure to unavoidable shock leads to a long-term deficit in avoidance learning [56], [57]. Interestingly, the LHb is necessary for the development of learned helplessness [58], and hyperactivity of the LHb, in particular increased excitatory drive onto LHb neurons projecting to the VTA [34], predisposes animals to develop learned helplessness [32]. Thus, an intriguing possibility is that inappropriate activation of the LHb during avoidance learning not only impaired the learning of the avoidance response, but also led to long-term brain changes that impaired future avoidance learning, in essence mimicking the learned helplessness effect.

A candidate for mediating the effect of LHb stimulation may be midbrain DA neurons. LHb stimulation inhibits DA neurons [19], [20], which are conventionally studied in the context of appetitive reward learning. But several studies argue that avoiding aversive stimuli is also rewarding [59], [60], [61], [62], [1]. Overall, approaching appetitive stimuli and avoiding aversive stimuli recruit similar brain circuits [63], [64], [23], [2], and DA plays a vital role in both [65], [3], [4]. For example, phasic DA bursting occurs upon the termination of aversive footshock [66] and fearful events [67], and DA signaling in the amygdala and striatum is vital for avoidance acquisition [68]. Therefore, increased DA signaling is expected to accompany the negative reinforcement event, which occurs when an animal performs the avoidance response that terminates the tone CS and prevents the expected shock US from occurring. Pairing LHb stimulation with this “relief” event presumably weakened this DA signal and DA-dependent processes for acquiring the avoidance response [69]. Supporting this hypothesis are findings that increased DA release occurs in the early, but not late, stages of avoidance learning [31], [70], [71]. Based on this finding, if LHb stimulation acts through a DA mechanism, it should have a behavioral impact when initiated early in training, but not when initiated late in training. As already discussed, this is exactly what was found.

In conclusion, our results support the emerging view of the LHb as an opponent player to the VTA in terms of biasing behavior toward a “no go” vs. “go” decision. That is, the LHb appears to suppress behaviors paired with its activation and to support passive avoidance [72], [73], [74]. In the case of two-way active avoidance learning, the influence of the LHb “anti-reward” signal appears to manifest only in the early stages of training, when there is a conflict between actively avoiding shock in the current chamber (“go”) vs. passively avoiding the chamber previously paired with shock (“no go”). In this case, the “go” decision is the adaptive one, and our results demonstrate how inappropriate or pathological activation of the LHb during this stage could lead to sustained maladaptive behavior in these types of challenges. This, together with other evidence, suggests a potential etiological role of LHb hyperactivity in depression, which is characterized by cognitive indecision and psychomotor retardation.
